# Experimental Syphilis

**Published:** 1905-10-21

**Authors:** 


### EXPERIMENTAL SYPHILIS.

Dr. Elie Metchnikoff has recently reviewed the present state of affairs as regards the experimental inoculation of syphilis which has been practised for some while past at the Pasteur Institute in Paris. The concise summary given in the "Medical Magazine" for August of Dr. Metchnikoff's address, states that fourteen chimpanzees have been successfully in-

ARMY MEDICAL LIBRARY

oculated on the skin or mucous surfaces, but that all attempts to produce the disease by peritoneal or subcutaneous contamination have been unsuccessful. The incubation period in the successful cases averaged thirty-one days, the variation in exceptional instances being as great as the fifteenth day in one and the forty-ninth day in another.

The first indication of a successful inoculation is a slight elevation of the skin at the seat of infection, followed on the succeeding day by a mild degree of redness and desquamation. In its further progress the local lesion resembles a human chancre; the primary lesion lasts about six or eight weeks and leaves an unpigmented scar. Chimpanzees present an enlargement of the lymphatic glands serving the affected site, just as is the case with men, but the presence or absence of a specific secondary roseola cannot be affirmed with confidence, since these apes are exceedingly prone even under normal conditions to roseolæ of various kinds. They develop, however, papules and ulcers which are certainly specific, since they are inoculable.

Of these fourteen chimpanzees all developed chancres, but in seven cases only was there any evidence of secondary lesions. When they did appear, the latter was first noted on or about the twenty-ninth day. Inoculations were attempted on seventy-nine monkeys, of which forty-six developed a primary sore without secondary manifestations. The incubation period was shorter here than in the case of the anthropoid apes, averaging twenty-five days. The younger the monkey the more refractory is it to the infection.

The syphilitic virus will not pass through a Berkfeld filter, and retains its virulence even when mixed with strong glycerine solution. Re-inoculation was successfully achieved as late as the tenth day after the first successful attempt. The spirochæte pallida was first seen by Bordet of Brussels in a chancre and mucous patches, but only on one occasion. In January, 1905, Siegel of Berlin stained it, and his results were confirmed by Schaudinn. This observer, working with Hoffman, found the spirochæte in the primary sores and mucous patches of twenty-six cases, and later in the inguinal glands and spleen also.

In malignant syphilis other organisms are at work besides the specific one. In the case of a chimpanzee dying of malignant syphilis death was due to a pneumococcal septicæmia. The specific organism has been further observed in the liver, spleen, and unbroken skin of sufferers from hereditary syphilis. But it has never been observed in any condition except the syphilis of men, apes, and monkeys. The spirochæte has so far resisted all efforts directed towards its artificial cultivation. An analogy exists between syphilis and relapsing fever, which, it will be remembered, is also dependent upon infection with a spirochæte, in the fact that both affect monkeys, and that in both a condition of endo-choroiditis is a common feature.

